# Histopathological analysis of vesicular and bullous lesions in Kaposi sarcoma

**DOI:** 10.1186/1746-1596-7-101

**Published:** 2012-08-15

**Authors:** Nilüfer Onak Kandemir, Figen Barut, Banu Doğan Gün, Nilgün Solak Tekin, Sevinç Hallaç Keser, Şükrü Oğuz Özdamar

**Affiliations:** 1 Department of Pathology, Karaelmas University, Faculty of Medicine, Zonguldak, Turkey; 2 Department of Dermathology, Karaelmas University, Faculty of Medicine, Zonguldak, Turkey; 3 Department of Pathology, Lütfi Kirdar Kartal Training and Research Hospital, Istanbul, Turkey

**Keywords:** Kaposi sarcoma, Bullous Kaposi sarcoma, Histological variants, Lymphangioma-like Kaposi sarcoma

## Abstract

**Background:**

In this study, the clinical and morphological features of vesiculobullous lesions observed in Kaposi sarcoma are analyzed, and the features of bullous Kaposi sarcoma cases are emphasized.

**Methods:**

A total of 178 biopsy materials of 75 cases diagnosed as classic-type cutaneous Kaposi sarcoma were reviewed. Twenty-five cases showing vesiculobullous features were included in the study. Tumor, epidermis, dermis, and clinical data regarding these cases was evaluated.

**Results:**

Vesicular changes were observed in 21 (12%) out of 178 lesions of the 75 cases, while bullous changes were present in only 4 (2%). In all cases where vesicular and bullous changes were detected, tumor, epidermis, and dermis changes were similar. All cases were nodular stage KS lesions, whereas hyperkeratosis and serum exudation in the epidermis, marked edema in the dermis, and enlarged lymphatic vessels and chronic inflammatory response were observed.

**Conclusions:**

Our findings suggest that changes in vascular resistance occurring during tumor progression are the most important factors comprising vesiculobullous morphology.

**Virtual slides:**

The virtual slide(s) for this article can be found here: http://www.diagnosticpathology.diagnomx.eu/vs/1646397188748474

## Background

Kaposi sarcoma (KS) is composed of four different epidemiological forms—classic, endemic, iatrogenic, and AIDS-associated (Acquired Immune Deficiency Syndrome) etiology comprising HHV-8 (Human Herpes Virus-8) [1]. The most common epidemiological form is the classic type. KS lesions are markedly different in terms of clinical picture and histopathological features, therefore many morphological variants have been defined, e.g., usual [UKS], anaplastic, telangiectatic, lymphangiectatic, hyperkeratotic, micronodular, keloidal, intravascular, pyogenic granuloma-like [PGLKS], lymphangiectatic [LKS], lymphangioma-like [LLKS], etc. UKS is the most common histological subtype, and the progression of the lesions is characterized by an increase in spindle cells [2-7].

Bullous KS [BKS] defines KS lesions in which a bullous appearance is clinically dominant. Bullous changes are rare in KS, but they are important in that they may be confused with other vesiculobullous skin disorders. The evolutionary mechanisms of bullous lesions in KS are still controversial, including whether or not they should be assessed as a different morphological subgroup [7-21].

In this study, the clinical and morphological features of vesiculobullous lesions observed in classic KS are analyzed, and the features of bullous KS lesions are emphasized in association with the relevant literature.

## Methods

### Sample selection

The study sample included 178 classic cutaneous KS cases in 75 patients who were diagnosed in the pathology departments of the University of Karaelmas, and Dr. Lütfi Kırdar Training and Research Hospital between 2001 and 2010. The clinical data was obtained from patient files. There was no history of immunosuppressive drug use, transplantation, or human immunodeficiency virus-1 [HIV-1] infection in any of the patients. All patients with KS were HIV-1 seronegative. All of the cases were classified as classic KS.

The study was performed in accordance with the Helsinki Declaration, and the privacy of the patients was protected through the decoding of data, according to the privacy regulations of the Zonguldak Karaelmas University Hospital (Zonguldak, Turkey), and Dr. Lütfi Kırdar Training and Research Hospital (İstanbul, Turkey).

### Histopathological evaluation

The histopathological diagnoses of all KS cases were confirmed through the examination of archived hematoxylin and eosin (H&E) slices, and immunohistochemical survey results. Histological sections of biopsy materials were assessed in terms of the properties of the tumors (histological type and stage), epidermis (atrophy, hyperkeratosis, papillomatous, vesicle and/or bulla, ulcer), and dermis (edema, inflammatory response, lymphangiectasia). Features were graded semi-quantitatively between 0–3 in a grading of histopathological findings, i.e., 0 – negative, 1 – mild, 2 – moderate, 3 – marked.

Blisters are fluid-filled cavities located within or beneath the epidermis. Blisters equal to or greater than 5 mm are called bullae, whereas blisters smaller than 5 mm are called vesicles. We also used these criteria to differentiate between vesicles and bullae [22].

The histological progression of KS lesions is composed of three phases, which can also be detected clinically: early (patch), intermediate (plaque), and late stages (nodule). The following criteria have been used to determine the histological stages of lesions:

Early phase: Lesions which are composed of inflammatory cells and newly-formed vascular structures, and contain no spindle cells.

Intermediate phase: Lesions with scattered spindle cells and cleft-like structures filled with erythrocytes, in addition to neoplastic vascular structures.

Late phase: Lesions in which spindle cells form nodular structures and blood-filled pseudovascular structures [1,2].

### Immunohistochemical analysis

Immunohistochemical analysis with endothelial indicators (CD31, CD34, D2-40) and HHV-8 (latent nuclear antigen-1 [LNA-1]) monoclonal antibodies were performed for confirmation of the histopathlogical diagnosis in each case. Immunohistochemical studies were performed using archival materials. A set of 4-μm-thick tissue sections was prepared from formalin-fixed and paraffin-embedded blocks, deparaffinized in xylene, rehydrated, and microwaved for 10 min at 30% power in a citrate buffer (pH 6.0) for antigen retrieval. Endogenous peroxidase activity was blocked using 0.3% hydrogen peroxide. The slides were then incubated at room temperature for 1 hour with D2-40 (1:100 dilution, Clone D2-40; BioCare Medical, Concord, CA, USA), CD31 (1:50 dilution, Clone JC/7A; NeoMarkers, Freemont, CA, USA), CD34 (1:100 dilution, Clone QBEnd/10; NeoMarkers), and HHV-8 (LNA- 1) (1:50 dilution, Clone 13B10; Novocastra, Newcastle, UK), using the biotin-streptavidin complex-diaminobenzidine (BSA-DAB) technique for detection. Negative controls were processed in parallel without primary antibodies. The D2-40 immunoreactivity of lymphatic endothelial cells, and the CD31 and CD34 immunoreactivity of capillaries in the upper dermis were used as internal positive controls.

## Results

**Figure 1:**
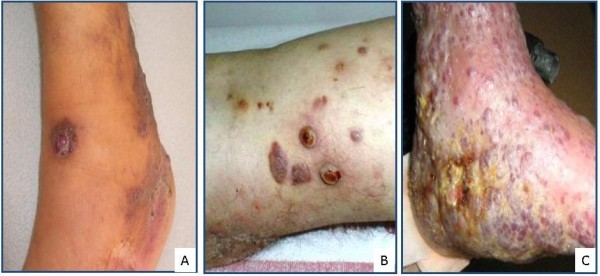
Different clinical appearances of lesions in bullous KS: (A) focal vesiculobullous changes in lesions of plaque and nodular stage, (B) bulla structures showing ulceration in nodular stage lesions, (C) sponge-like vesiculobullous lesions in a generalized KS case are observed.

Vesiculobullous histomorphology was observed in 25 (14%) out of 178 lesions of 75 cases. Vesicular formation was detected in 21 (12%) and bullous formation was detected in 4 (2%) cases in microscopic evaluation. Clinical vesicular changes were not detected in any of lesions in which vesicular formation microscopically whereas bullae were detected clinically in all lesions in which bullous formation was detected microscopically (Figure 1).

**Figure 2:**
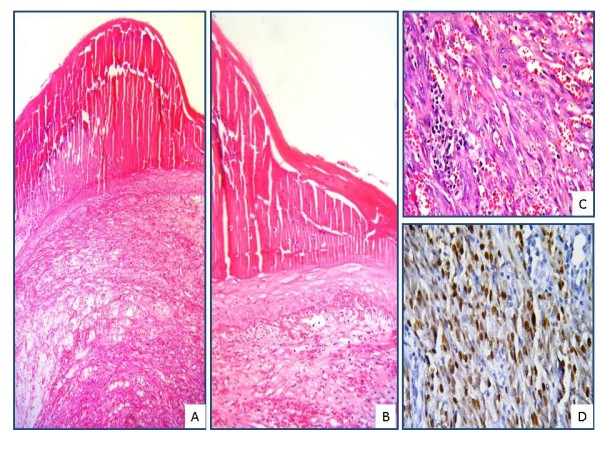
Hyperkeratosis (A) and serum exudation within keratin layer in epidermis and enlarged lymphatic vessels (B) in superficial dermis are observed in bullous KS (A-B; H&E, X400).

**Figure 3:**
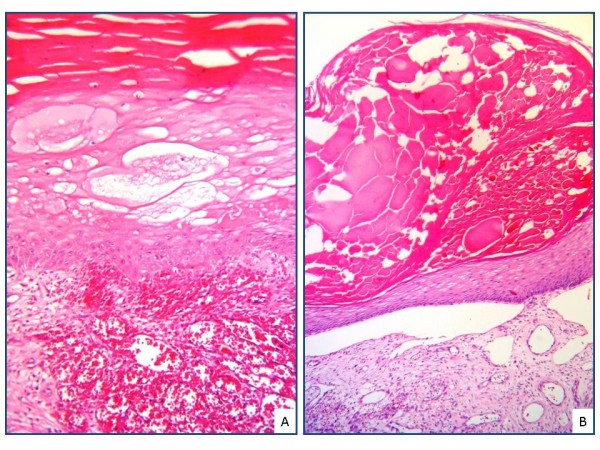
Intraepidermal (A) and subepidermal (B) splitting, superficial ulceration and marked dermal edema are observed in bullous KS (A-B; H&E, X200).

KS cases showing vesicular changes were present in 15 male (71%) and 6 female (29%) patients, and the mean age was found to be 72.50 ± 11.09 (min. 35–max. 84). Of all the lesions, 11 were seen in the lower extremities (52%), while 6 were in the upper extremities (29%) and 4 on the trunk (19%). All lesions were nodular stage KS lesions and, when epidermal changes were analyzed, hyperkeratosis was detected in 100% of the cases, serum exudation into the keratin layer in 64%, intraepidermal splitting in 84%, subepidermal splitting in16%, ulceration in 10%, papillomatous changes in 10%, and a rise in pigmentation on the basal membrane in 5%. Marked dermal edema and enlarged lymphatic vessels were observed in all cases, while chronic-type inflammatory response was seen in 86%. In cases in which ulceration was observed within the epidermis, neutrophil infiltration was detected in varying amounts, in addition to chronic-type inflammatory response (Table 1, Figure 2). When the histological subgroups of tumors were analyzed, 4 cases (19%) were assessed as LKS, 16 cases (76%) as UKS, and 1 case (5%) as PGLKS (Figure 3).

**Table 1:** Clinicopathological features of Kaposi sarcoma cases showing vesicular morphology (n = 21)

				**n**	**%**
	Age, mean ( min.- max.)			72.5 (35–84)	
Clinical features	Sex	Female		6	29
		Male		15	71
		Head&Neck		0	0
	Location	Trunk		4	19
		Extremities		17	81
	Histological sub-type	UKS		16	76
		LKS		4	19
		PGLKS		1	5
	Histological stage	Patch		0	0
		Plaque		0	0
		Nodular		21	100
		Hyperkeratosis		21	100
Histological features		Papillomatosis		2	10
		Atrophy		0	0
	Epidermis	Pigmentation		1	5
		Ulceration		5	24
		Vesicle	IE	17	84
			SE	4	16
		Edema		21	100
	Dermis	Fibrosis		0	0
		Inflammation		18	86
		Lymphangiectasia		21	100

KS: Kaposi sarcoma, UKS: Usual KS, LKS: Lymphangiectatic KS, PGLKS: Pyogenic granuloma-like KS, IE: Intraepidermal, SE: Subepidermal.

**Figure 4:**
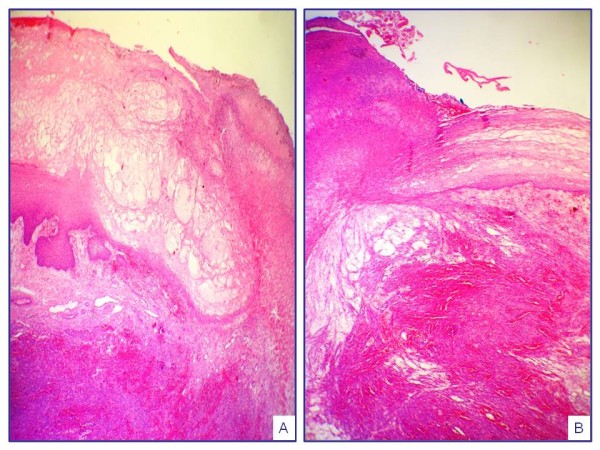
**Microscopic appearances of a bullous KS: intraepidermal bulla structure and serum exudation are observed within squamous epithelium of a nodular stage KS (A-B; H&E, A, X50, B, X100).** Nuclear immunoreaction with HHV-8 ( LNA-1) is observed in a tumor tissue composed of extravasated erythrocytes and spindle cells (C; H&E, X400, D; ABC-DAB, X400).

**Figure 5:**
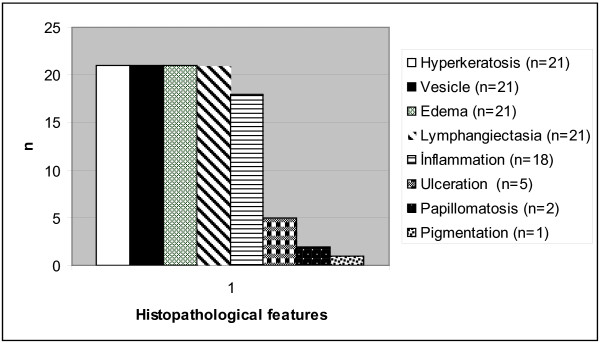
**Histopathological properties of Kaposi sarcoma cases showing vesiculer pattern (n = 21).**

**Figure 6:**
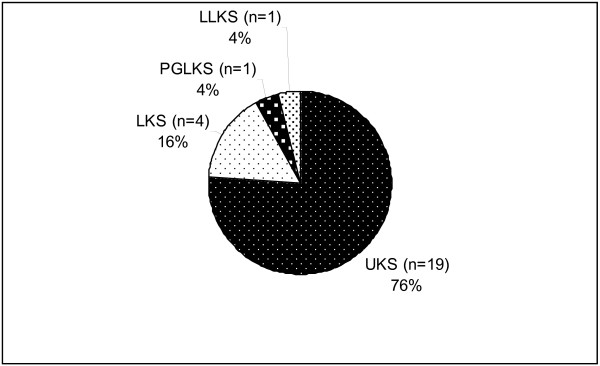
**Histological component of Kaposi sarcoma cases showing vesiculobullous pattern (n = 25).** KS: Kaposi sarcoma, UKS: Usual KS), LKS: Lymphangiectatic KS, LLKS: Lymphangiom- like KS, PGLKS: Pyogenic granuloma-like KS.

Bullous morphology was observed both clinically and histopathologically in 4 cases (16%), and these cases were evaluated as BKS. In cases in which bulla morphology, hence named BKS, was observed clinically, the mean age was found to be 66.75 ± 10.14 (min. 57-max. 72), and the female:male ratio was 1:3. The lesions were observed on the lower extremities (n = 2), the upper extremities (n = 1), and the head and neck region (n = 1). All cases were found to be nodular stage lesions, and serum exudation into the keratin layer was detected in 4 cases, intraepidermal (IE) and subepidermal (SE) bulla were detected in 3 and 1 cases, respectively, and ulceration in 2 cases. Papillomatosis was observed in 1 case and pigmentation increase in the basal layer was observed in 2 cases. Atrophy in the epidermal layer was not displayed in any of the cases. Marked edema, enlarged lymphatic vessels, and chronic-type inflammatory response were detected in all cases when the dermal properties were analyzed (Table 2, Figure 4, 5 and 6).

**Table 2:** Clinicopathological features of Kaposi sarcoma cases showing bullous morphology (n = 4)

**Clinicopathological features**			**Cases**			
			**1.**	**2.**	**3.**	**4.**
	Age		72	68	70	57
Clinical features	Sex		M	M	F	M
	Location		Hand	Leg	Food	Neck
	Histological stage		Nodular	Nodular	Nodular	Nodular
	Histological component		UKS	LLKS	UKS	UKS
		Hyperkeratosis	2+	2+	3+	1+
	Epidermis	Papillomatosis	0	0	1+	0
		Atrophy	0	0	0	0
Histological features		Pigmentation	1+	1+	0	0
		Ulceration	2+	1+	0	0
		Bull	IE	IE	IE	SE
		Edema	3+	3+	3+	3+
	Dermis	Fibrosis	0	0	0	0
		Inflammation	3+	2+	2+	1+
		Lymphangiectasia	3+	3+	3+	2+

KS: Kaposi sarcoma, UKS: Usual KS, LLKS: Lymphangiom-like KS, PGLKS: Pyogenic granuloma-like KS, IE: Intraepidermal, SE: Subepidermal, F: Female, M: Male.

The lesion cells of all KS cases showed various degrees of CD31, CD34, D2-40 (cytoplasmic), and HHV-8 (LNA-1) (nuclear) immunoreactivities. The non-neoplastic lymphatic endothelium, lymphangioma-like channels, proliferative capillaries, and spindle cells were reactive with D2-40, whereas the non-neoplastic blood vessels and thick-walled, mature vascular structures were non-reactive. The reaction was positive in non-neoplastic blood vessels with CD31 and CD34, while the non-neoplastic lymphatic vessels were non-reactive. There was a reaction with CD31 in all cellular components forming KS. There was a homogenous reaction with CD34 in neoplastic capillary structures, and spindle cells. A diffuse, strong nuclear reaction was observed with HHV-8 (LNA-1) in neoplastic vascular structures and spindle cells forming KS.

## Discussion

KS is classically characterized by blue-purple colored vascular lesions [1-5]. It is rare that these lesions demonstrate vesiculobullous morphology. Although vesiculobullous changes can be detected in different subgroups of KS, cases in which bullous changes dominate clinically are named bullous KS [5-21]. The clinical appearance of lesions in bullous KS can show a tight or collapsed bulla structure, ulcerated blister, or sponge-like properties. Marked dermal edema, intraepidermal or subepidermal splitting, and serum exudation in the epidermis can be seen histopathologically in these lesions. Bullous KS has the ability to imitate other vesiculobullous diseases clinically [7-22].

Bullous lesions in KS were first defined by Ronchese and Kern in 1957 [11]. To date, 30 reported cases with different terminologies have been available (Table 3) [5-21]. Cossu et al. reported bullous KS frequency at 4% in epidemic KS cases [13]. Notwithstanding, in our series composed of classic KS cases, vesiculobullous changes were observed in 14% of all patients, whereas only 2% of these met the diagnostic criteria of bullous KS. Our findings were consistent with those in the literature, and suggest that bullous morphology is a rare finding among KS cases. 

**Table 3:** Bullous Kaposi sarcoma: Review of the English-language literature

**Refence**	**N**	**Sex (n)**	**Age**	**Nomenclature**	**E. Type**	**Location**	**H. Stage**	**Clinical apperance**	**Association**	**Behavior**	**Year**
**Ronchese et al.**[11]	2	M	68-66	Cystic, LLKS	Classic	Lower extremity	Nodular	Bulla-like	-	Generalized/slow progresion	1957
**Gange et al.**[21]	3	F (1)	55-72 (63)	LLKS	Classic	Lower and	Nodular	Bulla-like (1),	-	Generalized/slow	1979
		M (2)				upper extremity		Papule (1)		progresion	
								Plaque (n = 1)			
**Leibowitz et al.**[18]	1	M	34	LLKS	-	Lower extremity	Nodular	Bulla-like	Lymphedema	Aggressive	1980
**Recth et al.**[17]	1	F	-	Bullous	Classic	Lower extremity	Nodular	Bulla-like, Vesicular	-	-	1986
**Bossuyt et al.**[19]	1	M	-	LLKS	AIDS	Lower and upper extremity	Nodular	Bulla-like	Lymphedema	Generalized/slow progresion	1995
**Perrot et al.**[14]	1	M	-	Bullous	AIDS	-	-	Bulla-like	Lymphedema	Aggressive	1995
**Noel et al.**[20]	1	M	-	LLKS	AIDS	Lower extremity	Nodular	Sponge-like	-	Generalized/slow progresion	1997
**Cossu et al.**[13]	7	F (1)	59-80 (70.3)	LLKS	Epidemic	Lower extremity (6)	Nodular	Bulla-like (3)	-	Aggressive (1)	1997
		M (6)				Upper extremity (1)		Sponge-like (4)		Generalized/	
										slow progresion (5)	
										Localized/ slow progresion (1)	
**Borroni et al.**[8]	1	F	82	Bullous	Classic	Lower extremity	Nodular	Bulla-like	-	-	1997
**Cottoni et al.**[10]	6	M (4)	56-80 (68.5)	LLKS (4)	Classic	Lower and	Nodular	Ulcer (2),	Lymphedema (4)	Aggresive (1)	1997
		F (2)		Regular (2)		upper extremity (2)		Bulla-like (1)			
						Lower extremity (4)		Serohematic loss (3)			
**Davis et al.**[12]	4	M	35	Bullous	AIDS	Lower extremity, scrotum	Nodular	Bulla-like	Lymphedema	Aggresive	2000
**Atillasoy et al.**[16]	1	M	41	Bullous	AIDS	Lower extremity	Nodular	Bulla-like, necrotic	Lymphedema	Aggresive	2001
**Torchia et al.**[9]	1	M	98	Pseudobullous	Classic	Lower extremity	Nodular	-	Lymphedema	Generalized/slow progresion	2008
**Present series**	4	M (3)	57-72 (66.7)	Bullous	Classic	Lower and	Nodular	Ulcer (2), bulla-like	-	Localized/	
		F (1)				upper extremity (3)		(4), serohematic crust		slow progresion (3)	
						Head &Neck (1)				Aggresive (1)	

*Age: Min.-Max. (mean), LLKS:* Lymphangioma-like Kaposi sarcoma, *E. Type: Epidemiological type ,H. stage: Histological stage, F: Female, M: Male, AIDS:****Acquired Immune Deficiency Syndrome.***

Grayson et al. reported that bullous changes were seen more frequently among classic KS cases [7]. When the clinical features of reported bullous classic KS cases were analyzed, it was seen that there was a higher number of older male patients and that lesions were largely found in the lower extremities. However, AIDS-related bullous KS cases can appear at earlier ages, and systemic involvement plus an aggressive clinical course might be observed in these cases [14]. In our study, it was found that 75% of bullous KS patients were male, and that the age range varied between 57 and 72. Fifty percent of these lesions were seen in the lower extremities, while 25% were in the upper extremities, and 25% in the head and neck region. In our series, it was detected that the demographic data of bullous KS cases was similar to that of other classic KS cases.

Blister formation in skin lesions develops by various mechanisms. The best-known mechanisms are spongiosis, achantholysis, ballooning degeneration, cytolysis, and basal membrane destruction. Among the mechanisms leading to blister formation are extracellular and intracellular edema, physical and chemical agents, infections, and immunological mechanisms. Spongiosis is the disintegration of keratinocytes because of accumulation of extracellular fluid in the epidermis. As a result, fluid-filled vesicles and bullae are formed in some cases. Spongiotic changes are accompanied by dermal edema, lymphatic dilatation, and perivascular inflammation [22].

Various theories regarding the etiopathogenesis of vesiculobullous lesions in KS have been suggested. Some researchers advocate that these lesions develop over chronic lymphedema ground, and that it is a specific entity, similar to lymphangioma circumscriptum [6,9,16]. However, some researchers have proposed that these dermal vascular changes are secondary to the local effects of tumor tissue and cause vesiculobullous morphology [7,9,14]. Factors inducing the development of bullous lesions in KS include the corruption of lymphatic drainage by tumor tissue, lymphaticovenous shunts formed on neoplastic vascular canals, lymphovascular invasion of tumor cells, the involvement of regional lymph nodes, and/or cytokine release. All of these factors can cause the development of stasis in cutaneous lymphatic vessels, dermal edema, and hence the formation of intraepidermal/subepidermal bulla secondary to absorption of lymphatic liquid by the epidermis [1,14-17,22].

In our series, all of the lesions showing bullous pattern were nodular stage lesions, and marked edema plus enlarged lymphatic vessels were observed within the papillary dermis. Intraepidermal and subepidermal bullae were detected in 3 and 1 cases, respectively. In all cases, serum exudation within the keratin layer was observed. Lymphedema was not detected clinically in any of the cases. Our findings suggest that lymphangiectasia and peritumoral edema secondary to tumor play a vital role in the etiology of bullous KS.

Hyperkeratosis is frequently observed, and in some cases, necrosis plus ulceration can be seen on the bulla ceiling in bullous KS cases. Dermal edema and enlarged lymphatic vessels are prominent in bullous KS cases, and fibrosis can be observed in those patients who receive radiotherapy [8-13]. In our study, hyperkeratosis was observed in all bullous KS cases, and ulceration was detected in two cases. Edema, enlarged lymphatic vessels, and chronic-type inflammatory response in varying degrees were observed in the dermis of all cases. Our findings show that histological findings accompanying the most frequently-found in bullous KS include hyperkeratosis, dermal edema, and lymphatic ectasia.

Cossu et al. reported that bullous changes are more frequent in KS cases showing lymphangioma-like features [13]. However, Cottoni et al. advocate that bullous changes cannot be related to any specific histological subgroup [10]. Although a large portion of cases reported in literature showed lymphangioma-like histological properties, bullous morphology can also be observed in pyogenic granuloma-like, lymphangiectatic, or usual-type KS cases. When the histological subgroups of KS cases with vesicular changes were analyzed in our study, 76% of all lesions showed usual-type properties, while 19% showed lymphangiectatic-type and 5% pyogenic granuloma-like properties. In one and three out of four cases assessed as bullous KS, lymphangiectatic- and usual-type KS properties dominated, respectively.

Vesicular changes (n = 21, 12%) were more common than bullous changes (n = 4, 2%) in KS lesions in our study. No vesicular formation was observed clinically in lesions in which microscopic vesicle formation was observed. On the other hand, a clinical bullous formation was observed in cases with microscopic bullous formation. Vesicle and bulla differentiation is associated with blister size. Growth in vesicle size by fluid accumulation results in the formation of bullae [22]. Thus, in our study, vesicles may not have been detected clinically due to the smaller vesicle size in cases with microscopic vesicles. Similar histopathological features of lesions with vesicular and bullous changes in our study suggest that these lesions represent different phases of the same process.

## Conclusions

In conclusion, the results of our study found that the demographic data of KS cases, which demonstrated a bullous pattern, was similar to that of other classic KS cases. Histologically, these lesions were closely associated with high stage, marked dermal edema and enlarged lymphatic vessels. Our findings support the idea that the vesiculobullous patterns observed during KS develop secondary to tumorogenesis. Therefore, the term “bullous KS” should be used as a clinical definition, and it should be remembered that it may be present in different histological subtypes. Clinical and histopathological recognition of vesiculobullous changes in KS is important so that accurate data can be assimilated for further studies.

## Abbreviations

KS: Kaposi sarcoma; HHV-8: Human herpesvirus 8; AIDS: Acquired Immune Deficiency Syndrome; PGLKS: Pyogenic granuloma-like KS; LLKS: Lymphangiom-like KS; UKS: Usual KS; BKS: Bullous KS; HIV-1: Human Immunodeficiency Virus-1; H&E: Hematoxylin and eosin; F: Female; M: Male; IE: Intraepidermal; SE: Subepidermal; LNA-1: Latent nuclear antigen-1; BSA-DAB: Biotin-Streptavidin complex- Diaminobenzidine.

## Competing interests

The authors declare that they have no competing interests.

## Authors’ contributions

NOK conducted the design of the study, performed microscopic evaluation, and drafted the manuscript. FB and NST participated in the design of the study and performed the selection of appropriate cases and data collection and helped to draft the manuscript. BDG and SHK participated in the design of the study and immunohistochemical evaluation. ŞOÖ conceived of the study, and participated in its design and coordination and helped to draft the manuscript. SHK participated in the design of the study and performed statistical analysis. All authors read and approved the final manuscript.
